# Temporomandibular Joint Pathology of Wild Carnivores in the Western USA

**DOI:** 10.3389/fvets.2021.657381

**Published:** 2021-04-08

**Authors:** Siobhan S. Rickert, Philip H. Kass, Frank J. M. Verstraete

**Affiliations:** ^1^Department of Surgical and Radiological Sciences, School of Veterinary Medicine, University of California, Davis, Davis, CA, United States; ^2^Department of Population Health and Reproduction, School of Veterinary Medicine, University of California, Davis, Davis, CA, United States

**Keywords:** temporomandibular joint, osteoarthritis, wildlife, dental pathology, dental abrasion

## Abstract

Skull specimens from: southern sea otter (*Enhydra lutris nereis*), Eastern Pacific harbor seal (*Phoca vitulina)*, California sea lion (*Zalophus californianus)*, northern fur seal (*Callorhinus ursinus*), walrus (*Odobenus rosmarus*), polar bear (*Ursus maritimus)*, North American brown bear (*Ursus arctos)*, American black bear *(Ursus americanus)*, California mountain lion (*Puma concolor couguar*), California bobcat (*Lynx rufus californicus)*, gray fox (*Urocyon cinereoargenteus)*, kit fox (*Vulpes macrotis)*, and gray wolf (*Canis lupus)* (*n* = 5,011) were macroscopically examined for dental and temporomandibular joint (TMJ) pathology. The presence of temporomandibular joint osteoarthritis (TMJ-OA) varied across species: 4.1% of southern sea otter, 34.5% of harbor seal, 85.5% of California sea lion, 20% of northern fur seal, 60.5% of walrus, 9.2% of polar bear, 13.2% of North American brown bear, 50% of American black bear, 20.9% of California mountain lion, 0% of California bobcat and gray fox, 6.3% of kit fox, and 11.6% of gray wolf specimens had lesions consistent with TMJ-OA. TMJ-OA was significantly more prevalent in males than females in walrus, North American brown bear, polar bear, American black bear, and California mountain lion (*p* < 0.001, *p* = 0.005, *p* = 0.005, *p* = 0.002, and *p* = 0.004, respectively). No other species showed a sex predilection. Adult specimens were significantly more affected with TMJ-OA than young adults in the harbor seal, fur seal, walrus (all *p* < 0.001), and kit fox (*p* = 0.001). Gray wolf and American black bear young adults were significantly (*p* = 0.047 and *p* < 0.001) more affected by TMJ-OA than adults. Of the 13 species analyzed, only three species, namely the harbor seal, northern fur seal, and polar bear, had a significant increase in the prevalence of TMJ-OA if their teeth had attrition and abrasion (*p* < 0.001, *p* < 0.001, and *p* = 0.033, respectively). TMJ-OA can lead to morbidity and mortality in wild animals, but its etiology is not yet fully understood.

## Introduction

The temporomandibular joint (TMJ) is an anatomical trait that is unique to the Mammalia class (1). The TMJ is a synovial joint made up of the mandibular head of the condylar process of the mandible and the mandibular fossa of the squamous portion of the temporal bone that are covered by a fibrocartilaginous layer (2–5). It is separated by an articular disc, creating a dorsal and ventral compartment in the joint. The articular surface is comprised of cartilage with an overlying fibrous component from the periosteum. During mastication the mandible is the mobile component of the bony components of the TMJ while all other components remain immobile (3). When masticatory muscles contract during mastication the TMJ is a load-bearing structure (1, 6).

**Figure 1:**
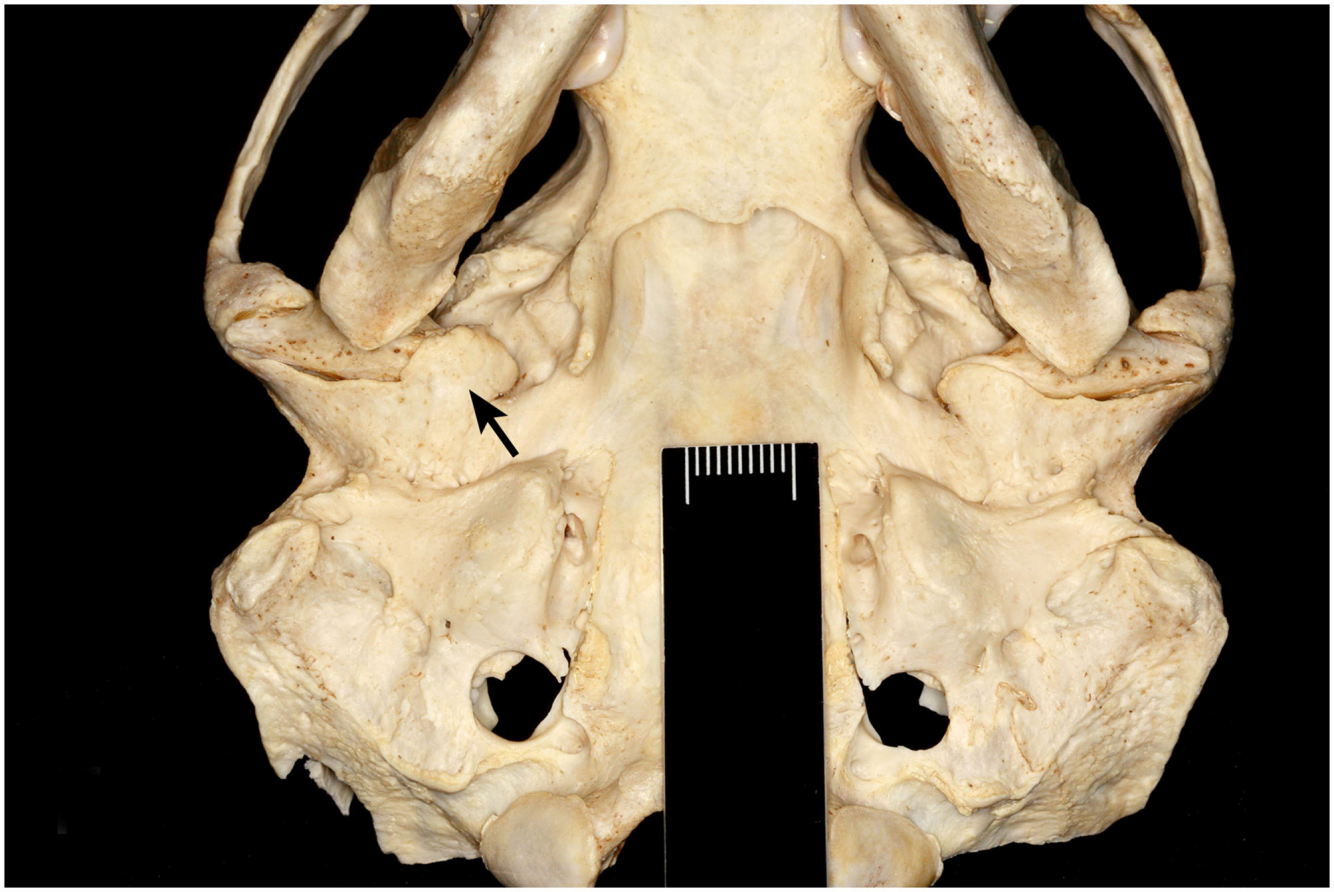
Ventral view of severe TMJ-OA of a male southern sea otter (*Enhydra lutris nereis*). Bony proliferation at the right retroarticular process (arrow) encircles the mandibular head, resulting in an inability to disarticulate the joint. Scale, 10 mm.

**Figure 2:**
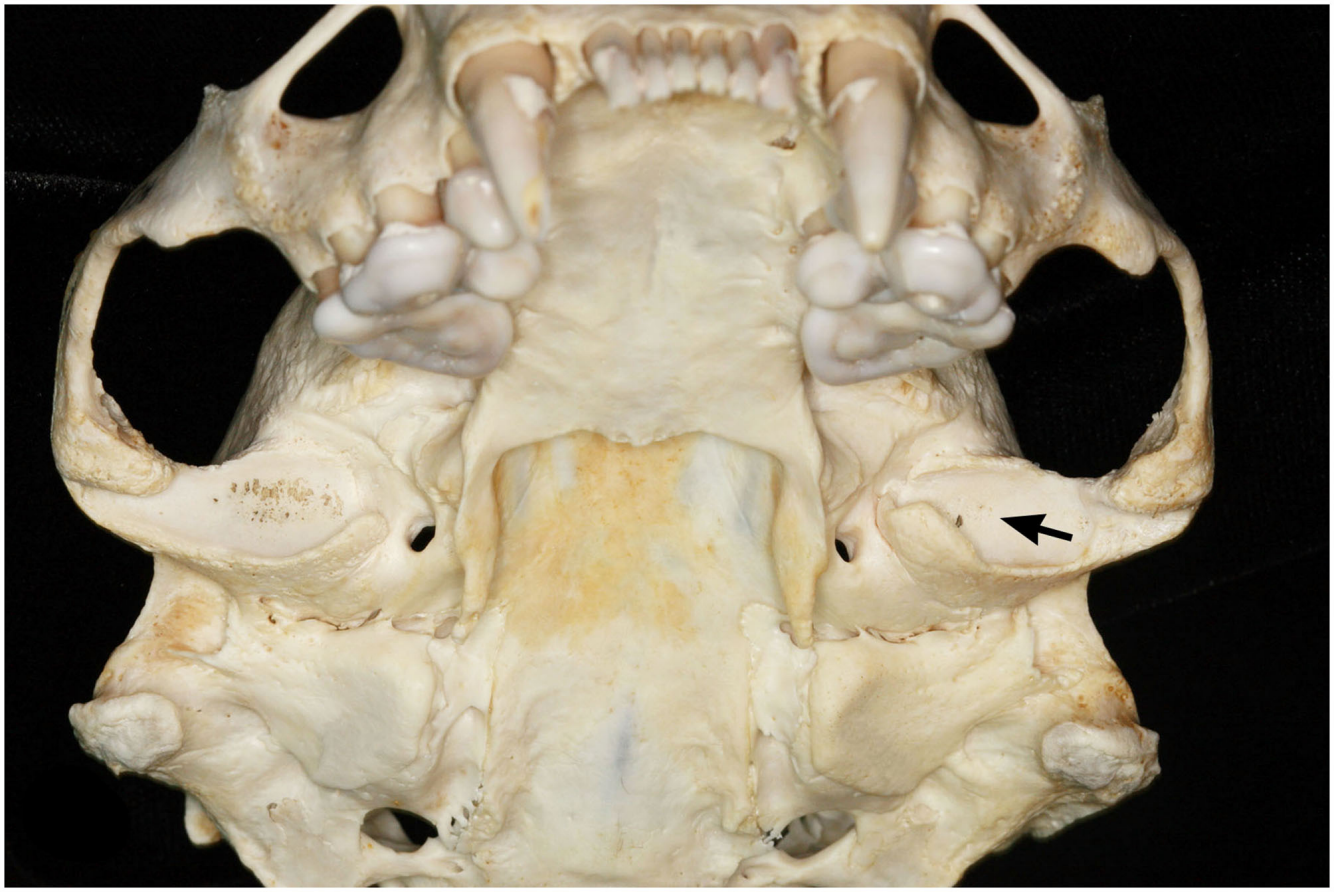
Ventral view of bilateral TMJ-OA of a female southern sea otter (*Enhydra lutris nereis*). The left mandibular fossa has mild OA as evidenced by mild subchondral bone erosion (arrow). The right mandibular fossa has severe OA as evidenced by more pronounced subchondral bone erosion and porosity.

**Figure 3:**
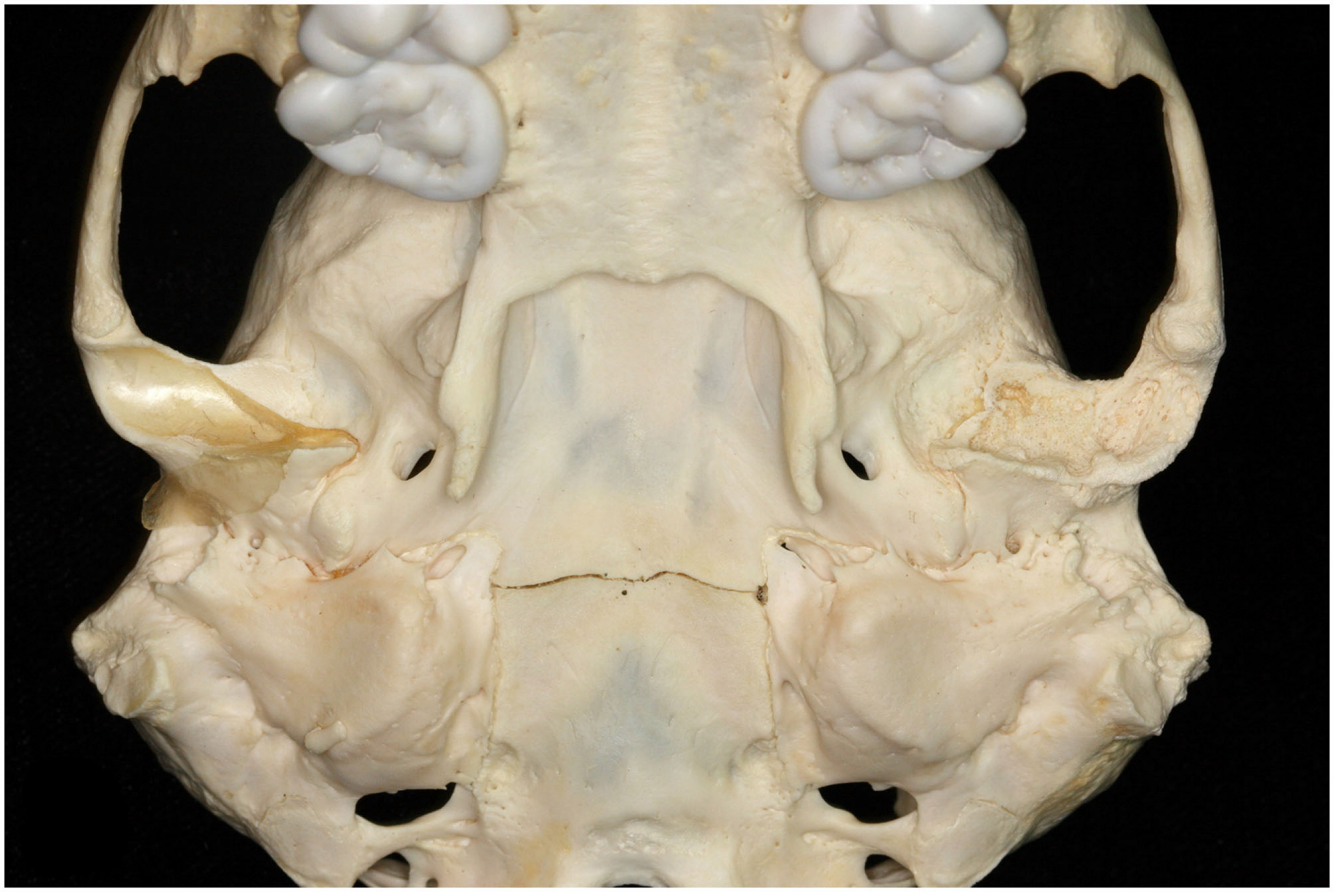
Ventral view of unilateral TMJ-OA at the left mandibular fossa of a female southern sea otter (*Enhydra lutris nereis*). Note the absence of articular surface and severe destruction of the subchondral bone.

Understanding TMJ pathology is integral to understanding a specific animal species' health. A functional TMJ is critical to an animal's ability to forage and express behaviors necessary for survival. TMJ pathology can lead to morbidity and, if severe, mortality of wild animal populations (Figures 1–3).

**Figure 4:**
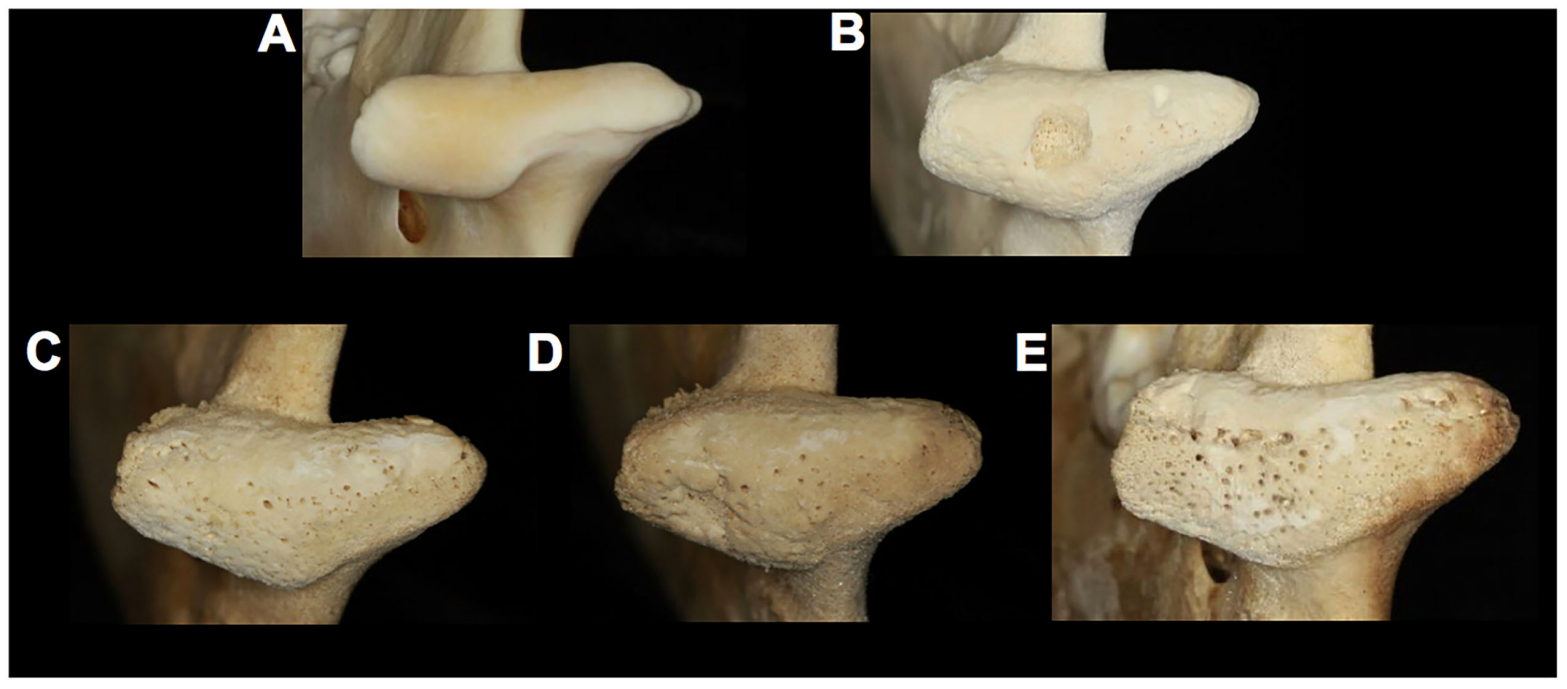
Comparison of gray wolf (*Canis lupus)* temporomandibular joints. **(A)** a healthy right mandibular head (UAM115902, unknown sex) with diseased right mandibular heads displaying **(B)** a severe central OCD-like lesion (UAM 70583, male) and **(C)** mild OA (UAM 87890, female), **(D)** moderate OA (UAM 87884, female) and **(E)** severe OA of the entire mandibular head (UAM 102040, unknown sex).

In health, the TMJ is critical to normal dental occlusion and the mandibular movement that allows mastication by redistributing the stress of these motions. Both of which are key components for survival (2, 7). TMJ osteoarthritis or osteoarthrosis (TMJ-OA) (depending on if there is an inflammatory origin) commonly occurs when the normal remodeling process caused by mechanical stress on the joint is overwhelmed, the adaptive capacity is exceeded, and the remodeling becomes pathologic. TMJ-OA is associated with pain and characterized by deterioration and abrasion of the articular cartilage and local thickening and remodeling of the underlying bone (Figure 4). Although the etiology of TMJ-OA is variable and unknown, it is believed to be the end result of many different pathogeneses; e.g., internal derangement of the disc, trauma, neoplasia, hormonal or metabolic changes, unbalanced loading of force or overloading of the joint, and malocclusion (6, 8).

In human medicine, TMJ disorders affect up to 25% of the population, although not all people get treatment (9). TMJ disorders disproportionately affect females, with two to eight times more females affected than males (2). Although TMJ disorders are generally considered degenerative, the majority of humans presenting to hospitals are between 20 and 50 years old (2). It has been proposed that patients with bruxism or attrition are more likely to acquire TMJ-OA, but as the body of scientific literature published on this subject grows, the associations between attrition and TMJ disorders remain unclear (10, 11).

There is interspecies variation in the function and morphology of the TMJ that has evolved based on feeding mechanisms (1). All species in this study are present in the Western United States, inhabiting a variety of habitat ranges. The following marine mammals, harbor seal (*Phoca vitulina)*, California sea lion (*Zalophus californianus)*, northern fur seal (*Callorhinus ursinus*), and walrus (*Odobenus rosmarus*) are all carnivores with non-masticatory feeding behaviors, i.e., they swallow their food whole after an initial bite to capture their prey, or suction shellfish out of their shells (12, 13). The southern sea otter (*Enhydra lutris nereis)*, on the other hand, is a marine mammal that relies heavily on mastication, eating a wide variety of marine species including hard shelled invertebrates such as sea urchins, mussels, clams and crabs which can be chewed whole (14, 15). Of the three bear species in this study, the polar bear (*Ursus maritimus)* is carnivorous, but eats primarily blubber and soft foods requiring minimal mastication (16). The North American brown bear (*Ursus arctos)* and the American black bear *(Ursus americanus)* are omnivorous, with the brown bear tending to be more carnivorous and the black bear tending to be more herbivorous (17, 18). The fibrous components of plants require more extensive mastication than muscle or fat. The California mountain lion (*Puma concolor couguar*) and the California bobcat (*Lynx rufus californicus)* are both carnivores, and use their teeth mostly for cutting and ripping meat (19). Of the three canids studied (gray fox (*Urocyon cinereoargenteus)*, kit fox (*Vulpes macrotis)*, and gray wolf (*Canis lupus))*, the gray fox is the only omnivorous opportunistic feeder. The kit fox and gray wolf are both carnivorous, but the kit foxes' primary diet is composed of small mammals, while gray wolves typically hunt prey larger than themselves (20–22).

The aim of this study was to determine the prevalence of TMJ-OA in wild carnivora and to determine if there is an association between TMJ-OA and attrition and abrasion by examining skulls within museum collections.

## Materials and Methods

Since 2004 skull specimens (*n* = 5,011) from 13 species were macroscopically examined by a team of researchers from the University of California, Davis School of Veterinary Medicine for dental and temporomandibular joint pathology based on predefined criteria first used in (23–25) and subsequently used in (26–39). The 5,011 specimens were made up of 1,007 southern sea otters, 194 harbor seals, 1,085 California sea lions, 145 northern fur seals, 76 walruses, 249 polar bears, 204 North American brown bears, 348 American black bears, 91 California mountain lions, 277 California bobcats, 569 gray foxes, 559 kit foxes, and 207 gray wolves. The specimens were housed in museum collections at the Department of Ornithology and Mammalogy, California Academy of Science in San Francisco, Museum of Vertebrate Zoology at the University of California, Berkeley, and Department of Mammalogy, Museum of the North, University of Alaska, Fairbanks. The specimens were each labeled with a catalog number, date and location of collection, and sex and age status (adult, young adult, and juvenile) if known. Juveniles were determined based on mixed dentition or the presence of deciduous teeth and young adults were identified by incomplete closure of the sutures in the skull. Only specimens with permanent dentition were analyzed. The only species in which juveniles were examined was the California sea lion because juveniles have a complete permanent dentition. California sea lion neonates were excluded from this study due to mixed or deciduous dentition. The specimens were obtained via public donations (commonly trappers and hunters), government agencies, stranded carcass recovery, wildlife center donations, “by-caught” animals, and unique to Alaska, specimens were also obtained by hunting permits for subsistence and animals killed under the “Defense of Life and Property” laws.

From the species-specific data we focused on the relevant pathologic lesions to compare the prevalence, sex, and age predilections across the 13 species. The presence of TMJ-OA and attrition and abrasion along with the sex and age of the skulls affected by these lesions were compiled. Additionally, specimens with attrition and abrasion were cross-checked with the specimens with TMJ-OA lesions to compile a list of specimens affected by both pathologic processes in order to determine if there was a correlation between attrition and abrasion and the presence of TMJ-OA.

**Figure 5:**
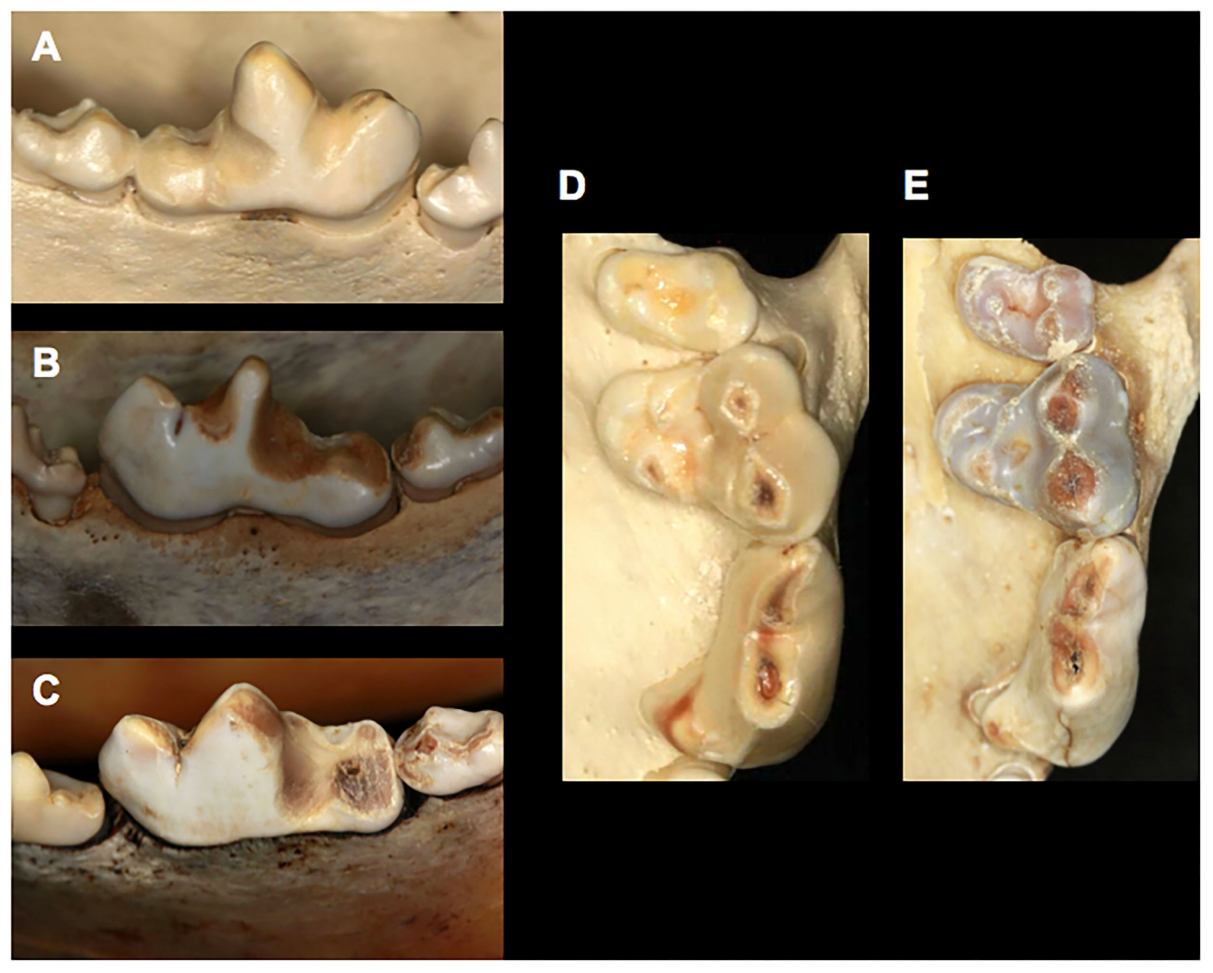
Different Gray wolf (*Canis lupus)* specimens with signs of attrition/abrasion: **(A)** mild attrition and abrasion of a right mandibular first molar tooth (UAM100504, unknown sex), **(B)** moderate attrition and abrasion of a left mandibular first molar tooth (UAM 24108, female), **(C)** severe attrition and abrasion of a left mandibular first molar tooth (UAM 21244, male), **(D)** minimal attrition and abrasion on a right maxillary second molar tooth and moderate attrition and abrasion on a maxillary first molar and fourth premolar tooth (UAM 70584, female) and **(E)** mild attrition and abrasion on a right maxillary second molar tooth, moderate attrition and abrasion on a maxillary first molar tooth and severe attrition and abrasion on a right maxillary fourth premolar tooth (UAM 101217, female).

To describe the wear of the teeth, each tooth was examined for attrition and abrasion. Attrition is defined as “the physiological wear of the teeth caused by the normal movement of teeth against one another or between food and teeth during mastication, hence the occlusal surfaces are commonly affected. Abrasion is defined as the wear due to an abnormal mechanical process.” (40). As it is difficult to differentiate the two processes during the macroscopic examination they were grouped together for the purpose of this study and are defined as “rounding or flattening of the cusp tip; exposure of dentine, with or without tertiary dentine formation” (24). For this study we used either the presence or absence of attrition and abrasion; in some of the species attrition and abrasion was recorded in stages of severity, but this method was not consistent across all species (Figure 5).

In order to examine skulls for TMJ pathology, the temporal fossa and the condylar process, including the mandibular head, were investigated on the right and left sides. Not all species examined in this study had scoring (mild, moderate, marked) recorded, therefore presence or absence of TMJ-OA was used. If none of the bony changes stated below were present, the joint was deemed normal, if any of these changes were present the skull was determined to have TMJ-OA. Mild TMJ-OA was scored if “any evidence of early lesions of periarticular new bone formation/osteophytes with minimal or no subchondral bone change.” Moderate TMJ-OA was defined as “periarticular new bone formation and/or subchondral bone changes were more pronounced.” Marked TMJ-OA was scored when “all of the previously described signs were present and more pronounced, or if subchondral bone lysis was present. A score of marked OA was also applied if partial or complete ankylosis was observed.” (25).

Investigators were trained to differentiate artificial damage, caused by the preparation and storage processes, from true pathology. Non-pathologic changes such as absent teeth without evidence of alveolar bone remodeling, fractures with sharp edges, or supraphysiological angles were all deemed to be caused by preparation or processing of the museum specimens. Skulls with considerable artifact were not included.

Thirteen species were divided into three groups based on their eating behavior. The first group was comprised of all non-masticatory marine mammals, including the Eastern Pacific harbor seal (*Phoca vitulina)*, California sea lion (*Zalophus californianus)*, northern fur seal (*Callorhinus ursinus*) and walrus (*Odobenus rosmarus*). The second group was made up of the polar bear (*Ursus maritimus)*, gray fox (*Urocyon cinereoargenteus)* and kit fox (*Vulpes macrotis)*, which were considered mild masticators. The final group contained the southern sea otter (*Enhydra lutris nereis*), North American brown bear (*Ursus arctos)*, American black bear *(Ursus americanus)*, California bobcat (*Lynx rufus californicus)*, gray wolf (*Canis lupus)* and California mountain lion (*Puma concolor couguar*), which are all heavily masticating species.

*Statistical Analysis*: In order to compare species, we applied new statistical methods to the previously reported data in the individual species papers (25–39). All of the specimen information, attrition and abrasion, TMJ pathology data, and skulls affected by both pathologies from all thirteen species were compiled. Pearson's chi-square test of homogeneity was used to compare the three species groups (defined by feeding behavior) with respect to the presence or absence of TMJ-OA. All other data was analyzed by Fisher's exact tests. Statistical analysis was performed using Stata 15.1/IC (StataCorp, College Station, TX). *P*-values <0.05 were considered statistically significant.

## Results

A summary of all thirteen species' percentages of skulls with pathologic lesions consistent with TMJ-OA, attrition and abrasion and skulls with concurrent TMJ-OA and attrition and abrasion are shown in Table 1.

**Table 1:** Percentage of skulls with TMJ-OA, attrition and abrasion, and skulls with both pathologies present.

**Species**	**% of skulls with TMJ-OA**	**% of skulls with Attrition and Abrasion**	**% of skulls with TMJ-OA and Attrition and Abrasion**
Southern sea otter (*Enhydra lutris nereis*)	4.1	92	3.7
Harbor seal (*Phoca vitulina)*	34.5	52	29
California sea lion (*Zalophus californianus)*	85.5	65.7	56.5
Northern fur seal (*Callorhinus ursinus*)	20	23.4	13.1
Walrus (*Odobenus rosmarus*)	60.5	92.1	57.9
Polar bear (*Ursus maritimus)*	9.2	16.5	3.2
North American brown bear (*Ursus arctos)*	13.2	100	13.2
American black bear *(Ursus americanus)*	50	97.1	48.3
California mountain lion (*Puma concolor couguar*)	20.9	93.4	19.8
California bobcat (*Lynx rufus californicus)*	0	85.2	0
Gray fox (*Urocyon cinereoargenteus)*	0	85.6	0
Kit fox (*Vulpes macrotis)*	6.3	90.5	6.3
Gray wolf (*Canis lupus)*	11.6	69	6.9

### Marine Mammals (Excluding the Polar Bear, Which Is Included in the Ursidae Section)

Southern sea otter (*Enhydra lutris nereis*)
  Of the 1,007 southern sea otter skulls examined 539 were males, 463 females, and five were of an unknown sex. Seven hundred thirty-six of the specimens were adults and 271 were young adults. Of the 1,007 southern sea otter skulls, 41 had TMJ-OA present (4.1%) and 929 specimens exhibited attrition and abrasion (92%). There was no sex or age predilection for skulls with TMJ pathology. There was no statistical significance between male and females with attrition and abrasion. Adults had significantly (*p* < 0.001) more attrition and abrasion present compared to young adults. Thirty-seven (3.7%) specimens had both TMJ-OA and attrition and abrasion present. Eight hundred ninety two of the 966 skulls without TMJ-OA had attrition and abrasion present, while 37 of the 41 skulls with TMJ-OA had signs of attrition and abrasion, meaning the proportion of specimens with attrition and abrasion was not significantly different between southern sea otters with and without TMJ pathology.
Eastern Pacific harbor seal (*Phoca vitulina)*
  One hundred ninety four specimens comprised of 89 males, 98 females, and five skulls of an unknown sex were examined. One hundred one of the specimens were adults and 93 were young adults. Sixty seven skulls (34.5%) had TMJ-OA and 101 skulls (52%) exhibited signs of attrition and abrasion. Adults were significantly (*p* < 0.001) more likely to be affected by both TMJ-OA and attrition and abrasion, compared to young adults. Neither males nor females were more affected by TMJ-OA or attrition and abrasion. Fifty seven (29%) of the skulls had both attrition and abrasion as well as TMJ-OA. The proportion of specimens with attrition and abrasion is significantly different (*p* < 0.001) between harbor seals with TMJ-OA vs. harbor seals without TMJ-OA. Thirty five percent (44/127) of specimens without TMJ pathology had attrition and abrasion, while 85% (57/67) of specimens with TMJ-OA had attrition and abrasion as well.
California sea lion (*Zalophus californianus)*
  The 1,085 skulls examined comprised 671 males, 405 females, and nine animals of unknown sex. Six hundred twenty seven were adults, 351 were young adults and 107 were juveniles. Of the 1,085 California sea lions examined 928 (85.5%) had TMJ-OA lesions and 713 (65.7%) were affected by attrition and abrasion. Neither age nor sex were significantly different for specimens with TMJ-OA. Both males and adults were significantly more affected (*p* = 0.008 and *p* < 0.001, respectively) by attrition and abrasion than females and young adults. Six hundred thirteen (56.5%) skulls examined had both TMJ-OA and attrition and abrasion present. One hundred of the 157 (63.7%) skulls without TMJ-OA had attrition and abrasion present, meaning there was no significant difference in the proportion of specimens with attrition and abrasion in California sea lions with, and without, TMJ pathology.
Northern fur seal (*Callorhinus ursinus*)
  Of the 145 specimens examined 71 were males, 56 were females and 18 were of an unknown sex, while 58 were adults and 87 were young adults. Twenty nine of the 145 skulls analyzed (20%) had TMJ-OA present and 34 (23.4%) of examined skulls had attrition and abrasion. Adults were significantly more likely than young adults to have TMJ-OA (*p* < 0.001) and attrition and abrasion (*p* < 0.001). There was no significant difference between males and females for both attrition and abrasion and TMJ-OA lesions. Nineteen (13.1%) of the specimens examined had both TMJ-OA and attrition and abrasion present. The proportion of specimens with attrition and abrasion was statistically significantly (*p* < 0.001) between northern fur seals with TMJ and northern fur seal without TMJ-OA. 12.9% (15/116) of specimens without TMJ pathology had attrition and abrasion, while 65.5% (19/29) of specimens with TMJ-OA had attrition and abrasion.
Walrus (*Odobenus rosmarus*)
  The 76 specimens were comprised of 45 males, 29 females and two skulls of unknown sex. Fifty eight of the skulls were adults and 18 were young adults. Of the 76 walrus skulls examined 46 specimens (60.5%) had TMJ-OA and 70 (92.1%) had attrition and abrasion present. Both TMJ-OA and attrition and abrasion were significantly more likely in adults than young adults (*p* < 0.001 and *p* = 0.025, respectively). TMJ-OA was found to be significantly (*p* < 0.001) more prevalent in males than females. Of the 76 specimens investigated, 44 (57.9%) had both TMJ-OA and attrition and abrasion. There was no significant difference between the specimens with attrition and abrasion and the presence of TMJ-OA. 86.6% (26/30) of the specimens without TMJ-OA had attrition and abrasion present, while 95.6% (44/46) of the specimens with TMJ-OA also had attrition and abrasion.

### Ursidae

Polar bear (*Ursus maritimus)*
  The 249 polar bear specimens included 126 males, 93 females, and 30 animals of an unknown sex. The distribution of adults and young adults were almost equal with 125 and 124, respectively. Twenty three (9.2%) of the 249 polar bear specimens had lesions consistent with TMJ-OA and 41 (16.5%) of the skulls has attrition and abrasion present. Males were significantly more likely to have TMJ-OA and attrition and abrasion (*p* = 0.005 and *p* = 0.009, respectively) than females. Adults were significantly (*p* = 0.039) more likely to have attrition and abrasion than young adults, but there was no significant difference present between ages for TMJ-OA. Eight (3.2%) of the skulls had both TMJ-OA and attrition and abrasion present. There was a significant (*p* = 0.033) increase in prevalence of TMJ-OA if the specimen's teeth were affected by attrition and abrasion. Fourteen point six percent (33/226) of specimens without TMJ pathology had attrition and abrasion, while 34.8% (8/23) of specimens with TMJ-OA had attrition and abrasion.
North American brown bear (*Ursus arctos)*
  Of the 204 North American brown bear specimens 99 were males, 87 were females, and 18 were unknown, 92 were adults and 112 were young adults. Thirteen point two percent (*n* = 27) of the skulls examined had TMJ-OA and 100% (*n* = 204) had attrition and abrasion. Males were significantly more affected by TMJ-OA (*p* = 0.005) than females, but there was no significant age difference. Additionally, there was no significant difference between age or sex for the skulls with attrition and abrasion. All 27 specimens affected with TMJ-OA also had attrition and abrasion present. The proportion of teeth with attrition/abrasion was not significantly different between those with and without TMJ pathology. One hundred percent of specimens with and without TMJ-OA had attrition and abrasion present.
American black bear *(Ursus americanus)*
  The 348 skulls examined included 125 males, 107 females, and 116 animals of an unknown sex. There were 284 adults and 64 young adults. One hundred seventy four (50%) had TMJ-OA present and 338 specimens (97.1%) had attrition and abrasion. Males were significantly (*p* = 0.002) more affected by TMJ-OA than females, but there was no sex predilection for attrition and abrasion. Young adults were significantly more affected (*p* < 0.001) than adults for TMJ-OA, but adults were significantly (*p* = 0.022) more affected by attrition and abrasion. One hundred sixty eight (48.3%) of the specimens had both TMJ-OA and attrition and abrasion present. The proportion of specimens affected by attrition and abrasion was not significantly different between those with and without TMJ-OA. Ninety seven point seven percent (170/174) of specimens without TMJ pathology had attrition and abrasion, while 96.5% (168/174) of specimens with TMJ-OA had attrition and abrasion.

### Felidae

California mountain lion (*Puma concolor couguar*)
  The 91 California mountain lions comprised 42 males, 34 females, and 15 of an unknown sex. In the collection there were 39 adults and 52 young adults. Nineteen (20.9%) skulls were affected by TMJ-OA and 85 (93.4%) skulls had signs of attrition and abrasion. Males were significantly (*p* = 0.004) more affected by TMJ-OA than females, but there was no significant difference between adults and young adults. Neither age grouping, nor sex, were found to have a significant difference for specimens affected by attrition and abrasion. All specimens with TMJ-OA were also affected by attrition and abrasion. Ninety one point six percent (66/72) of specimens without TMJ pathology had attrition and abrasion, while 100% (19/19) of specimens with TMJ-OA had attrition and abrasion. There was no significant difference in specimens affected by attrition and abrasion between skulls with TMJ-OA and without TMJ-OA.
California bobcat (*Lynx rufus californicus)*
  Of the 277 specimens examined, 128 were males, 114 females, and 35 were an unknown sex. There were 221 adults and 56 young adults. Two hundred thirty six (85.2%) of the specimens were affected by attrition and abrasion and none were affected by TMJ-OA. There was no significant difference between sex or age in the specimens affected by attrition and abrasion.

### Canidae

Gray fox (*Urocyon cinereoargenteus)*
  The 569 gray fox skulls examined included 261 males, 196 females, and 112 with sex unknown. There were 481 adults, 67 young adults, and 21 whose age was unknown. Of the 569 skulls examined, none of them were affected by TMJ-OA and 487 (85.6%) skulls were affected by attrition and abrasion. Adult skulls were significantly (*p* < 0.001) more affected with attrition and abrasion than young adults.
Kit fox (*Vulpes macrotis)*
  The 559 kit fox skulls consisted of 267 males, 248 females, and 44 animals of unknown sex. Four hundred thirty one skulls were adults and 128 skulls were young adult animals. Thirty five (6.3%) specimens were affected by TMJ-OA and 506 (90.5%) were affected by attrition and abrasion. Adults were significantly more affected by both TMJ-OA and attrition and abrasion (*p* = 0.001 and *p* < 0.001, respectively) than young adults. Female kit foxes were significantly (*p* = 0.011) more affected by attrition and abrasion than males. There was no significant difference between sexes for the skulls affected with TMJ-OA. All 35 of the specimens with TMJ-OA were found to also have attrition and abrasion. Eighty nine point eight percent (471/524) of specimens without TMJ pathology had attrition and abrasion, while 100% (35/35) of specimens with TMJ-OA had attrition and abrasion meaning there was no significant difference found between the specimens affected by attrition and abrasion between skulls with TMJ-OA and without TMJ-OA.
Gray wolf (*Canis lupus)*
  The 207 gray wolf specimens included 65 males, 104 females, and 38 unknown sex. There were 83 adults and 124 young adults. Twenty four (11.6%) of the 207 gray wolf specimens examined were affected by TMJ-OA and 143 (69%) specimens had attrition and abrasion present. Adults were significantly (*p* < 0.001) more affected with attrition and abrasion than young adults, but young adults were significantly (*p* = 0.047) more affected by TMJ-OA than adults. Neither TMJ-OA or attrition and abrasion was found to have significance between males and females. Fourteen (6.9%) of the skulls affected with TMJ-OA were also affected by attrition and abrasion, but there was no significant difference between specimens affected with attrition and abrasion and specimens with and without TMJ pathology. Seventy point five percent (129/183) of specimens without TMJ pathology had attrition and abrasion, while 58.3% (14/24) of specimens with TMJ-OA had attrition and abrasion.

### Age

Of all 13 animal species studied, adult eastern Pacific harbor seal, northern fur seal, walrus, and kit fox were significantly (*p* < 0.001, *p* < 0.001, *p* < 0.001, and *p* = 0.001, respectively) more affected than young adults with TMJ-OA. Adult Southern sea otter, eastern Pacific harbor seal, California sea lions, Northern fur seal, walrus, polar bear, American black bear, gray fox, kit fox, and gray wolf were significantly (*p* < 0.001, *p* < 0.001, *p* < 0.001, *p* < 0.001, *p* = 0.025, *p* = 0.039, *p* = 0.022, *p* < 0.001, *p* < 0.001, and *p* < 0.001, respectively) more affected with attrition and abrasion than young adults. In the American black bear and gray wolf, young adults were significantly (*p* < 0.001 and *p* = 0.047, respectively) more affected by TMJ-OA than adults (Table 2).

**Table 2:** Percentages of skulls with TMJ-OA divided by sex and age groupings.

**Species**	**% of females skulls with TMJ-OA**	**% of males skulls with TMJ-OA**	**% of adults skulls with TMJ-OA**	**% of young adults skulls with TMJ-OA**
Southern sea otter (*Enhydra lutris nereis*)	5.4	3.0	4.7	2.2
Harbor seal (*Phoca vitulina)*	29.6	40.4	54.4^*^	12.9^*^
California sea lion (*Zalophus californianus)*	83.6	86.7	86.6	82.9
Northern fur seal (*Callorhinus ursinus*)	28.6	16.9	37.9^*^	8.0^*^
Walrus (*Odobenus rosmarus*)	17.2^*^	86.6^*^	75.9^*^	11.1^*^
Polar bear (*Ursus maritimus)*	2.1^*^	12.7^*^	11.2	7.3
North American brown bear (*Ursus arctos)*	5.7^*^	20.2^*^	10.9	15.2
American black bear *(Ursus americanus)*	35.5^*^	56.8^*^	43.0^*^	81.2^*^
California mountain lion (*Puma concolor couguar*)	8.8^*^	38.1^*^	23.1	19.2
California bobcat (*Lynx rufus californicus)*	0	0	0	0
Gray fox (*Urocyon cinereoargenteus)*	0	0	0	0
Kit fox (*Vulpes macrotis)*	8.1	4.9	7.9^*^	0.8^*^
Gray wolf (*Canis lupus)*	9.6	15.4	6.0^*^	15.3^*^

* *indicates a significant (p < 0.05) difference between age or sex affected by TMJ-OA*.

### Sex

Males were significantly more affected by TMJ-OA than females in walrus (*p* < 0.001), polar bear (*p* = 0.005), North American brown bear (*p* = 0.005), American black bear (*p* = 0.002), and California mountain lion (*p* = 0.004). Males were significantly more affected by attrition and abrasion in California sea lions (*p* = 0.008), polar bear (*p* = 0.009). Female kit foxes were significantly (*p* = 0.011) more affected by attrition and abrasion than males (Table 2).

### Attrition and Abrasion and TMJ-OA

Of the 13 species analyzed, three species: the harbor seal, Northern fur seal and polar bear, had a significant increase in the prevalence of TMJ-OA if their teeth had attrition and abrasion (*p* < 0.001, *p* < 0.001, and *p* = 0.033, respectively).

### Feeding Behavior

Differences in TMJ-OA prevalence between the three species groupings were significantly different (*p* < 0.001). In non-masticatory marine mammals, mild masticators, and heavily masticating species the prevalences were 79.9, 4.2, and 13.7, respectively.

## Discussion

Attrition and abrasion are signs of wear, and therefore, point to use of the teeth (mastication) and work of the TMJ. Interestingly only three species, the harbor seal, Northern fur seal and polar bear, had a significant increase in the prevalence of TMJ-OA when their teeth had attrition and abrasion. This leads us to believe there is no consistent association between heavy chewing and TMJ-OA. This finding is consistent with inconclusive studies between dental wear and TMJ degeneration in the human literature (10, 11). In addition, the three species with increased prevalence are not heavily masticating species; the harbor seal and Northern fur seal swallow their food whole, while the polar bear eats mostly blubber (13, 16). This could mean most of the species studied have enough remodeling capacity to balance off the stress and compensation of the TMJ, thus avoiding TMJ-OA during normal mastication for the species. TMJ-OA is therefore more likely caused by another etiology, not normal mastication. Some possible etiologies for TMJ-OA could include genetic factors, feeding behaviors, endocrinopathies, neoplasia, trauma, anatomical structural differences, polyarthritis or a multifactorial disease (6, 8). Mastication on an abnormal joint that already has TMJ-OA might further exacerbate and worsen the severity of the disease process but is unlikely to be the sole cause of TMJ-OA in these wild carnivora species. Further studies into TMJ-OA in wild carnivora in live animals or on specimens with soft tissues present are needed to provide further insight into this disease etiology and its role in morbidity and mortality of these populations.

In 1936 Colyer published “Variations and Diseases of the Teeth of Animals,” in which museum specimens from the Odontological Museum of the Royal College of Surgeons of England were examined. While Colyer did discuss attrition and abrasion, there was no mention of TMJ pathology in his work (41). The first published cases of TMJ pathology in wild carnivores was discovered in Northern elephant seals (*Mirounga angustirostris*) in 2005. Of the 104 specimens examined, three had signs of TMJ-OA (28). Since 2004 a team of researchers at the University of California, Davis have collected data from 13 species from three museum collections: California Academy of Sciences, Museum of Vertebrate Zoology of the University of California Berkeley, and Museum of the North, University of Alaska, Fairbanks. In the 13 species analyzed in this study, a wide range of TMJ-OA prevalence was found: 4.1% of southern sea otter, 34.5% of harbor seal, 85.5% of California sea lion, 20% of northern fur seal, 60.5% of walrus, 9.2% of polar bear, 13.2% of North American brown bear, 50% of American black bear, 20.9% of California mountain lion, 0% of California bobcat and gray fox, 6.3% of kit fox, and 11.6% of gray wolf specimens.

Only five species of the 13 species examined, the walrus, North American brown bear, polar bear, American black bear, and California mountain lion had a sex predilection, with males being significantly more affected than females. None of the species were found to have females significantly more affected by TMJ-OA than males. These findings show that there is no consistent pattern of which sex is more affected by TMJ-OA across the 13 species in this study. This is a direct contradiction to human TMJ-OA literature in which females are two-eight times more likely to have TMJ disease (2). In human medicine a suggested estrogen receptor polymorphism related to the increased prevalence of TMJ disorders in females might explain the large discrepancy between humans and wild animals (42). Additionally, in some of these wild populations males behave very differently from females, particularly in fighting other males for mates or territories. As an example of behavior differences between sexes leading to a difference in prevalence of pathology males walruses are more likely to have tusk fractures than females (12). This behavioral variation may cause different biomechanical stress or forces on the TMJ that the body can no longer compensate for, and therefore could cause a significant difference in the prevalence of TMJ-OA between sexes. Interestingly, in every bear species we examined males were significantly more affected than females. Further studies into the way each sex utilized their TMJ would provide insight into this question.

Across species there was no clear age distribution for TMJ-OA. In four species, eastern Pacific harbor seal, northern fur seal, walrus, and kit fox, adults were significantly more affected. However, in two species, the American black bear and gray wolf, young adults were significantly more affected than adults. TMJ-OA occurring in an older population follows logically because it is considered an acquired lesion. The older the animal, the more likely it is to acquire TMJ-OA. This is at odds with humans findings where there is a clear pattern of people between the ages of 20–50 being more affected than other age groups (2). As this study only analyzed museum specimens, we do not know when symptoms of TMJ disease started in these animals. Additionally the exact ages of these specimens are not known, there could be a range of ages within each age grouping, having precise ages could provide more insight into the age of onset and possible causes of etiology. It would be possible to obtain a far more accurate understanding of the role of animal age affected by TMJ-OA via a longitudinal study of live patients.

When we divided our 13 species into three groups based on how much mastication they utilize during normal feeding behaviors (non-masticatory, light mastication and heavy mastication), there was significant difference in the prevalence of TMJ-OA between three groups. Further studies into the ecological feeding behaviors and presence of TMJ-OA would be interesting to determine if anatomical changes in maxillofacial structures, such as size of musculature or location of the condylar process, affect the prevalence of TMJ-OA among species. For example, 85.5% of California sea lion skulls are affected by TMJ-OA. It has been postulated that the strong bite force required to catch their prey, their large oral aperture, and their low condylar process, may have an effect on acquiring TMJ-OA (43).

Due to the nature of this study we only had access to the hard tissues and could not analyze the soft tissue structures associated with the TMJ. Analyzing the disc for internal derangement, soft tissue trauma, and other possible derangements, could be critical to understanding the underlying etiology of TMJ-OA in wild carnivores. Additionally, we did not have consistent data on the severity of the lesions across all species which could provide insight into morbidity in these animals. Analysis of the entire skeleton, to access if there is polyarthritis present, would also be beneficial. [In the seven complete southern sea otter skeletons with TMJ-OA six showed signs of polyarthritis within the appendicular and vertebral joints (44)].

There was no clear association between attrition and abrasion, sex, or age across all wildlife species examined here. It is clear the signalment and current understanding of human TMJ disorders, while important to draw knowledge from, do not completely cross over into our wild animal populations, particularly in relation to sex and age. One can assume that TMJ-OA will contribute to morbidity and, in severe cases, mortality of these wild animals, therefore understanding its etiology and the consequences of this disease will be helpful on both an individual and population health level (45). Although we are still not certain what the etiology of the TMJ-OA was in the wild species examined, it is clear that normal mastication is not a consistent cause of TMJ-OA across species. Additional research into the etiology, and possible predisposing factors to the development of TMJ-OA in wild carnivores will be beneficial to better understanding of wildlife pathology.

## Data Availability Statement

Publicly available datasets were analyzed in this study. The original data are published in the literature and references are provided.

## Author Contributions

SR and FV: conceived and designed study. SR: compiled and analyzed data. PK: statistical analysis. SR, FV, and PK: drafted and edited manuscript. All authors contributed to the article and approved the submitted version.

## Conflict of Interest

The authors declare that the research was conducted in the absence of any commercial or financial relationships that could be construed as a potential conflict of interest.

## References

[1] Herring SW. TMJ anatomy and animal models. J Musculoskeletal Neuronal Intertact. (2003) 3:391–407. Available online at: http://www.ismni.org/jmni/pdf/14/38HERRING.pdf PMC2821032 15758330

[2] Murphy MK MacBarb RF Wong ME Athanasiou KA. Temporomandibular joint disorders: a review of etiology, clinical management, and tissue engineering strategies. Int J Oral Maxillofac Implants. (2013) 28:393–414. 10.11607/jomi.te20 24278954 PMC4349514

[3] Athanasiou KA Almarza AJ Detamore MS Kalpakci KM. Tissue engineering of temporomandibular joint cartilage. M C Tissue Eng. (2009) 1:1–122. 10.2200/S00198ED1V01Y200906TIS00217822153

[4] Lantz GC Arzi B. Temporomandibular joint dysplasia. In: Verstraete FJM Lommer MJ Arzi B editors. Oral and Maxillofacial Surgery in Dogs and Cats. 2nd ed. St. Louis, MO: Elsevier (2020). p. 361–7.

[5] Lantz GC Arzi B. Fractures and luxations involving the temporomandibular joint. In: Verstraete FJM Lommer MJ Arzi B editors. Oral and Maxillofacial Surgery in Dogs and Cats. 2nd ed. St. Louis, MO: Elsevier (2020). p. 368–76.

[6] Tanka E Detamore MS Mercuri LG. Degenerative disorders of the temporomandibular joint: etiology, diagnosis, and treatment. Crit Rev Oral Biol Med. (2008) 87:296–307. 10.1177/154405910808700406 18362309

[7] Fanghanel J Gedrange T. On the development, morphology and function of the temporomandibular joint in light of the orofacial system. Ann Anat. (2007) 189:314–9. 10.1016/j.aanat.2007.02.024 17695983

[8] Wang XD Zhang JN Gan YH Zhou YH. Current understanding of pathogenesis and treatment of TMJ osteoarthritis. J Dent Res. (2015) 94:666–73. 10.1177/0022034515574770 25744069

[9] Solberg WK Woo MW Houston JB. Prevalence of mandibular dysfunction in young adults. J Am Dent Assoc. (1979) 98:25–34. 282342 10.14219/jada.archive.1979.0008

[10] Yadav S. A study on prevalence of dental attrition and its relation to factors of age, gender and to the signs of TMJ dysfunction. J Indian Prosthodont Soc. (2011) 11:98–105. 10.1007/s13191-011-0076-7 22654349 PMC3120954

[11] van't Spijker A Kreulen CM Creugers NHJ. Attrition, occlusion, (dys)function, and intervention: a systematic review. Clin Oral Implants Res. (2007) 18(Suppl. 3):117–26. 10.1111/j.1600-0501.2007.01458.x 17594376

[12] Fay FH. Ecology and biology of the Pacific walrus, Odobenus rosmarus divergens liliger. Washington (DC): United States Fish and Wildlife Service (1981). p. 279.

[13] Reeves RR Stewart BS Clapham PJ Powell JA. National Audubon Society Guide to Marine Mammals of the World. New York, NY: Alfred A. Knopf, Incorporated (2002). p. 527.

[14] Murie OJ. Notes on the sea otter. J Mammal. (1940) 21:119–31. 10.2307/1374968

[15] Kenyon KW. The sea otter in the eastern Pacific Ocean. N Amer Fauna. (1969) 68:1–352.

[16] Slater GJ Figueirido B Louis L Yang P Van Valkenburgh B. Biomechanical consequences of rapid evolution in the polar bear lineage. PLoS ONE. (2010) 5:11. 10.1371/journal.pone.0013870 21079768 PMC2974639

[17] Pasitschniak-Arts M. Ursus arctos. Mamm Species. (1993) 439:1–10. 10.2307/3504138

[18] Larivière S. Ursus americanus. Mamm Species. (2001) 647:1–11. 10.1644/1545-1410(2001)647<0001:UA>2.0.CO;2

[19] Toon A Toon SB. Cats (Felidae). In: Hutchins M Kleiman DG Geist V McDade MC editors. Grzimek's Animal Life Encyclopedia. 2nd ed. Farmington Hills, MI: Gale Group (2003). p. 369–92.

[20] Fritzell EK Haroldson KJ. Urocyon cinereoargenteus. Mamm Species. (1982) 189:1–8. 10.2307/3503957

[21] McGrew JC. Vulpes macrotis. Mamm Species. (1979) 123:1–6. 10.2307/3504038

[22] Mech LD. Canis lupus. Mamm Species. (1974) 37:1–6. 10.2307/3503924

[23] Verstraete FJM van Aarde RJ Nieuwoudt BA Mauer E Kass PH. The dental pathology of feral cats on Marion Island, part I: congenital, developmental and traumatic abnormalities. J Comp Path. (1996) 115:265–82. 10.1016/S0021-9975(96)80084-3 8923237

[24] Verstraete FJM van Aarde RJ Nieuwoudt BA Mauer E Kass PH. The dental pathology of feral cats on Marion Island, part II: periodontitis, external odontoclastic resorption lesions and mandibular thickening. J Comp Path. (1996) 115:283–97. 10.1016/S0021-9975(96)80085-5 8923238

[25] Arzi B Winer JN Kass PH Verstraete FJM. Osteoarthritis of the temporomandibular joint in southern sea otters (*Enhydra lutris nereis*). J Comp Path. (2013) 149:486–94. 10.1016/j.jcpa.2013.03.009 23721871

[26] Aalderink MT Nguyen HP Kass PH Arzi B Verstraete FJM. Dental and temporomandibular joint pathology of the northern fur seal (*Callorhinus ursinus*). J Comp Path. (2015) 152:325–34. 10.1016/j.jcpa.2015.02.002 25824117

[27] Aalderink MT Nguyen HP Kass PH Arzi B Verstraete FJM. Dental and temporomandibular joint pathology of the Eastern Pacific harbour seal (*Phoca vitulina richardii*). J Comp Path. (2015) 152:335–44. 10.1016/j.jcpa.2015.02.003 25824118

[28] Abbott C Verstraete FJM. The dental pathology of northern elephant seals (*Mirounga angustirostris*). J Comp Path. (2005) 132:169–78. 10.1016/j.jcpa.2004.09.007 15737343

[29] Aghashani A Kim AS Kass PH Verstraete FJM. Dental pathology of the California bobcat (*Lynx rufus californicus*). J Comp Path. (2016) 154:329–40. 10.1016/j.jcpa.2016.03.001 27102444

[30] Aghashani A Kim AS Kass PH Verstraete FJM. Dental and temporomandibular joint pathology of the California mountain lion (*Puma concolor couguar*). J Comp Path. (2017) 156:251–63. 10.1016/j.jcpa.2016.11.269 28024874

[31] Clark EJ Chesnutt S Winer JN Kass PH Verstraete FJM. Dental pathology of the American black bear (*Ursus americanus*). J Comp Path. (2017) 156:240–50. 10.1016/j.jcpa.2016.11.267 27989366

[32] Döring S Arzi B Winer JN Kass PH Verstraete FJM. Dental and temporomandibular joint pathology of the grey wolf (*Canis lupus*). J Comp Path. (2018) 160:56–70. 10.1016/j.jcpa.2018.03.001 29729722

[33] Evenhuis JV Zisman I Kass PH Verstraete FJM. Dental pathology of the grey fox (*Urocyon cinereoargenteus*). J Comp Path. (2018) 158:39–50. 10.1016/j.jcpa.2017.11.002 29422314

[34] Sinai NL Dadaian RH Kass PH Verstraete FJM. Dental pathology of the California sea lion (*Zalophus californianus*). J Comp Path. (2014) 151:113–21. 10.1016/j.jcpa.2014.02.004 24725510

[35] Winer JN Arzi B Leale DM Kass PH Verstraete FJM. Dental and temporomandibular joint pathology of the walrus (*Odobenus rosmarus*). J Comp Path. (2016) 155:242–53. 10.1016/j.jcpa.2016.07.005 27530539

[36] Winer JN Arzi B Leale DM Kass PH Verstraete FJM. Dental and temporomandibular joint pathology of the polar bear (*Ursus maritimus*). J Comp Path. (2016) 155:231–41. 10.1016/j.jcpa.2016.07.004 27481648

[37] Winer JN Arzi B Döring S Kass PH Verstraete FJM. Dental and temporomandibular joint pathology of the North American brown bear (*Ursus arctos horribilis, Ursus arctos middendorffi and Ursus arctos sitkensis*). J Comp Path. (2017) 157:90–102. 10.1016/j.jcpa.2017.06.006 28942310

[38] Winer JN Liong SM Verstraete FJM. The dental pathology of southern sea otters (*Enhydra lutris nereis*). J Comp Path. (2013) 149:346–55. 10.1016/j.jcpa.2012.11.243 23348015

[39] Yanagisawa N Wilson RE Kass PH Verstraete FJM. Dental and temporomandibular joint pathology of the kit fox (*Vulpes macrotis*). J Comp Path. (2019) 167:60–72. 10.1016/j.jcpa.2019.01.001 30898300

[40] Sivapathasundharam B. Regressive alterations of the teeth. In: Shafer WG Hine MK Levy BM editors. A Textbook of Oral Pathology. Philadelphia, PA: Saunders (1983). p. 318–38.

[41] Colyer F. Variation and diseases of the teeth of animals. London: John Bale, Sons & Danielsson, Ltd. (1936) p. 758.

[42] Riberio-Dasilva MC Line SRP dos Santos MCLG Arthuri MT Hou W Fillingim RB. Estrogen receptor-α polymorphisms and predisposition to TMJ disorder. J Pain. (2009) 10:527–33. 10.1016/j.jpain.2008.11.012 19411060 PMC2749669

[43] Arzi B Murphy MK Leale DM Vapniarsky-Arzi N Verstraete FMJ. The temporomandibular joint of California sea lions (*Zalophus californianus*): Part 1 – Characterisation in health and disease. Arch Oral Biol. (2015) 60:208–15. 10.1016/j.archoralbio.2014.09.004 25451464

[44] Arzi B Leale DM Sinai NL Kass PH Lin A Verstraete FMJ. The temporomandibular joint of California sea lions (*Zalophus californianus*): Part 2 – osteoarthritic changes. Arch Oral Biol. (2015) 60:216–22. 10.1016/j.archoralbio.2014.09.005 25451465

[45] Arzi B Cissell DD Verstraete FJM Kass PH DuRaine GD Athanasiou KA. Computed tomographic findings in dogs and cats with temporomandibular joint disorders: 58 cases (2006-2011). J Am Vet Med Assoc. (2013) 242:69–75. 10.2460/javma.242.1.69 23234284 PMC3747040

